# Comparison and Identification of the Aroma-Active Compounds in the Root of *Angelica dahurica*

**DOI:** 10.3390/molecules24234352

**Published:** 2019-11-28

**Authors:** Die Hu, Junrui Guo, Ting Li, Mu Zhao, Tingting Zou, Huanlu Song, Aygul Alim

**Affiliations:** 1 Beijing Higher Institution Engineering Research Center of Food Additives and Ingredients, Beijing Technology and Business University (BTBU), Beijing 100048, China; tianshijie2525@163.com (D.H.); gjr990316@163.com (J.G.); liting120902@163.com (T.L.); zhaomu2985@163.com (M.Z.); songhl@th.btbu.edu.cn (H.S.); 2 Beijing Key Laboratory of Flavor Chemistry, Beijing Technology and Business University (BTBU), Beijing 100048, China; 3 College of Food Science and Pharmacy, Xinjiang Agriculture University, Xinjiang 830052, China; aygulalim@sina.com

**Keywords:** *Angelica dahurica* root, aroma-active compounds, solid-phase microextraction (SPME), gas chromatography-olfactometry-mass spectrometry (GC-O-MS), aroma extract dilution analysis (AEDA)

## Abstract

Solid-phase microextraction (SPME), purge and trap (P&T), stir bar sportive extraction (SBSE), and dynamic headspace sampling (DHS) were applied to extract, separate and analyze the volatile compounds in the roots of Hangbaizhi, Qibaizhi, and Bobaizhi and the GC-O-MS/MS (AEDA) was utilized for the quantification of key aroma compounds. Totals of 52, 54, and 43 aroma-active compounds extracted from the three samples by the four extraction methods were identified. Among these methods, the SPME effectively extracted the aroma compounds from the *A. dahurica*. Thus, using the SPME methods for quantitative analysis based on external standards and subsequent dilution analyses, totals of 20, 21, and 17 aroma compounds were detected in the three samples by the sniffing test, and sensory evaluations indicated that the aromas of *A. dahurica* included herb, spice, and woody. Finally, principal component analysis (PCA) showed that the three kinds *A. dahurica* formed three separate groups, and partial least squares discriminant analysis (PLS-DA) showed that caryophyllene, (−)-β-elemene, nonanal, and β-pinene played an important role in the classification of *A. dahurica*.

## 1. Introduction

*Angelica dahurica* (Baizhi) is a traditional herb in China, according to the location of origin the *A. dahurica* has 5 types: Hangbaizhi, Qibaizhi, Chuanbaizhi, Yubaizhi, and Bobaizhi [1]. The *Angelica dahurica* is a dual-purpose plant with medicinal and edible value. On the one hand, with the increasing health consciousness, *A. dahurica* will be increasingly popular, as it has anti-inflammatory, antioxidant, antibacterial, and anticancer properties [2,3,4,5]. Root of *Angelica dahurica* was defined in the 2010 edition of the China Pharmacopoeia (2010) [6] as the dry root of *Angelica dahurica* (Fisch.ex Hoffm.) Benth.et Hook.f. or *Angelica dahurica* (Fisch.ex Hoffm.) Benth.et Hook.f. var. formosana (Boiss.) Shanet Yuan. On the other hand, its edible parts are used as a spice for flavoring, seasoning, or imparting aroma. In previous work, out of the very few studies on the flavor of the root of *A. dahurica*, Li, Lu and Feng [7] use gas chromatography-mass spectrometry (GC-MS) to analyze the volatile components, such as 1,8-cineol, limonene and caryophyllene (SPME). Furthermore, the terpenes, α-limonene, 3-decene, and 1-*R*-α-pinene were identified in the volatile oil of *A. dahurica* [8], and Li et al. [9] found that dodecanal is the most abundant species in the volatile oil of Chuanbaizhi and Hangbaizhi and found that volatile oils have many different flavors.

However, a comprehensive analysis of the key aroma-active compounds of the root of *A. dahurica* has not been reported. In further investigations focusing on the flavor, some scholars have studied the aroma components of botanical seasonings [10,11,12,13]. Omar et al. [12] used dynamic headspace sampling-gas chromatography-mass spectrometry (DHS-GC-MS) to analyze the monoterpenoids in four aromatic plants [14]. Raffo et al. [13] identified the key aroma-active components in rocket leaves by combining headspace solid-phase microextraction (HS-SPME), stir bar sportive extraction (SBSE) with GC-MS, and gas chromatography-olfactometry (GC-O); they found that the most potent odor-active compounds identified included (*Z*)-3-hexenal, (*E*)-3-hexenal, (*Z*)-1,5-octadien-3-one, and 4-mercaptobutyl [15]. However, the summary on the characteristics of odorents on the different of types *A. dahurica*, especially, qualification and quantification on the aroma compounds was still fragmentary.

The main objectives of this study were, firstly, to compare the four headspace analysis methods (SPME, P&T, SBSE, and DHS) to identify the volatile flavor compounds from the three types of *A. dahurica*. Secondly, SPME-AEDA and flavor dilution (FD) factors were employed to identify and quantify ten key aroma-active compounds shared by the three types of *A. dahurica*. Finally, the *A. dahurica* samples were divided into three groups by principal component analysis (PCA), and partial least squares discriminant analysis (PLS-DA) was used to identify the aroma-active compounds that most strongly influenced the classification.

## 2. Results and Discussion

### 2.1. Comparison of Quantity of Aroma Compounds by the Different Extraction Methods

Three replicate experiments using the same sample were performed on the same day to test the repeatability of each method. The coefficients of variation (CV values) of the total ion counts for SPME, P&T, SBSE, and DHS are 1.40%, 5.65%, 6.76%, and 6.441%, respectively, indicating that SPME was more repeatable than the other three methods.

**Figure 1:**
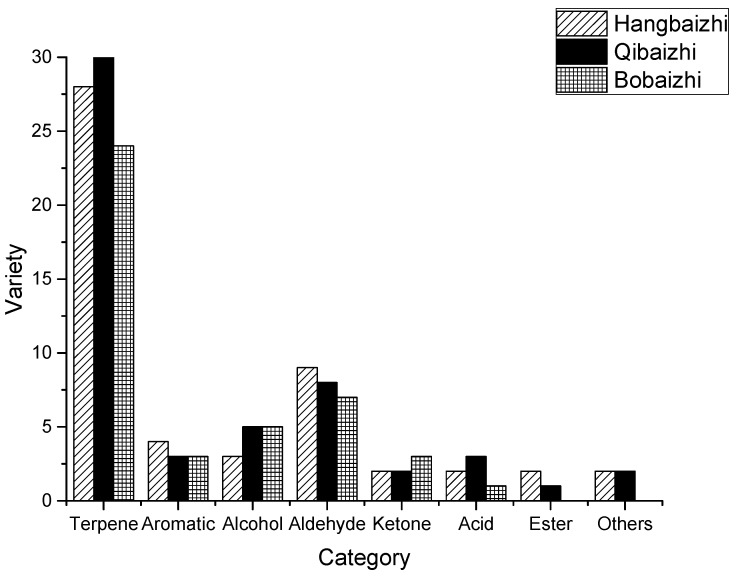
Types of flavor compounds extracted by SPME, P&T, SBSE, and DHS from Hangbaizhi, Qibaizhi and Bobaizhi.

The compounds extracted by the four headspace extraction methods and analyzed by GC-O-MS are shown in Table 1 and Figure 1. A total of 52, 54, and 43 aroma compounds were identified in the samples from Hangbaizhi, Qibaizhi, and Bobaizhi, respectively. These compounds included terpenes, aromatic species, alcohols, aldehydes, ketones, acids, and esters. Moreover, a total of 20, 21, and 17 aroma-active compounds were detected in the *A. dahurica* samples from Hangbaizhi, Qibaizhi and Bobaizhi, respectively, by the sniff test. These compounds contributed aroma characteristics of pine, camphor, woody, herb, citrus, spice, cucumber, sour, and so on. We also found that the most abundant type of compounds in all three types of *A. dahurica* were terpenes, with 28, 30, and 24 derivatives detected in Hangbaizhi, Qibaizhi, and Bobaizhi, respectively, followed by aldehydes with 9, 8, and 7 derivatives identified in Hangbaizhi, Qibaizhi and Bobaizhi, respectively. Among these, 1,8-cineol, limonene and caryophyllene have been detected previously from the root of *A. dahurica* [7], to use gas chromatography-mass spectrometry (GC-MS) by SPME.

The fifty-two aroma compounds detected by GC-O-MS in the sample from Hangbaizhi are listed in Table 1. The compounds represented a variety of smells, such as pine, camphor, woody, spice, herb, citrus, sour, and grass. Among these compounds, the content of terpenes, the main contributors to the fragrance of certain plants, was the highest. Terpenes may substantially contribute to the aroma, and thus the flavor, of Hangbaizhi. The content of aldehydes (9 derivatives) was second highest. The aromas of aldehydes can be described as fatty, cut-grass-like, and citrusy. Because aldehydes usually have low odor thresholds, they may contribute to the overall odor of *A. dahurica*. Terpenes (followed by aldehydes) were also the most abundant compounds extracted from Qibaizhi and Bobaizhi. Nineteen terpenes and 6 aldehydes were common aroma components among the three kinds of *A. dahurica*, which may cause the *A. dahurica* samples from different locations to have similar odor characteristics. Qibaizhi and Bobaizhi contained more kinds of alcohols than Hangbaizhi; however, due to their low contents or high thresholds, few alcohols were detected during the sniff tests. Aromatic species, ketones, acids, and esters detected in the *A. dahurica* samples may also contribute to their overall odor.

### 2.2. Comparison of Quality of Aroma Compounds by the Different Extraction Methods

As shown in Table 1, 31 common aroma compounds were identified in all three samples. A total of 34, 38, and 26 aroma compounds were extracted from Hangbaizhi, Qibaizhi, and Bobaizhi, respectively, by SPME; 15, 17, and 15 aroma compounds were extracted by P&T; 29, 38, and 22 aroma compounds were extracted by SBSE, while 29, 33, and 23 aroma compounds were extracted by DHS. The variety of compounds extracted by P&T was the lowest, whereas those extracted by the other methods were not significantly different.

**Figure 2:**
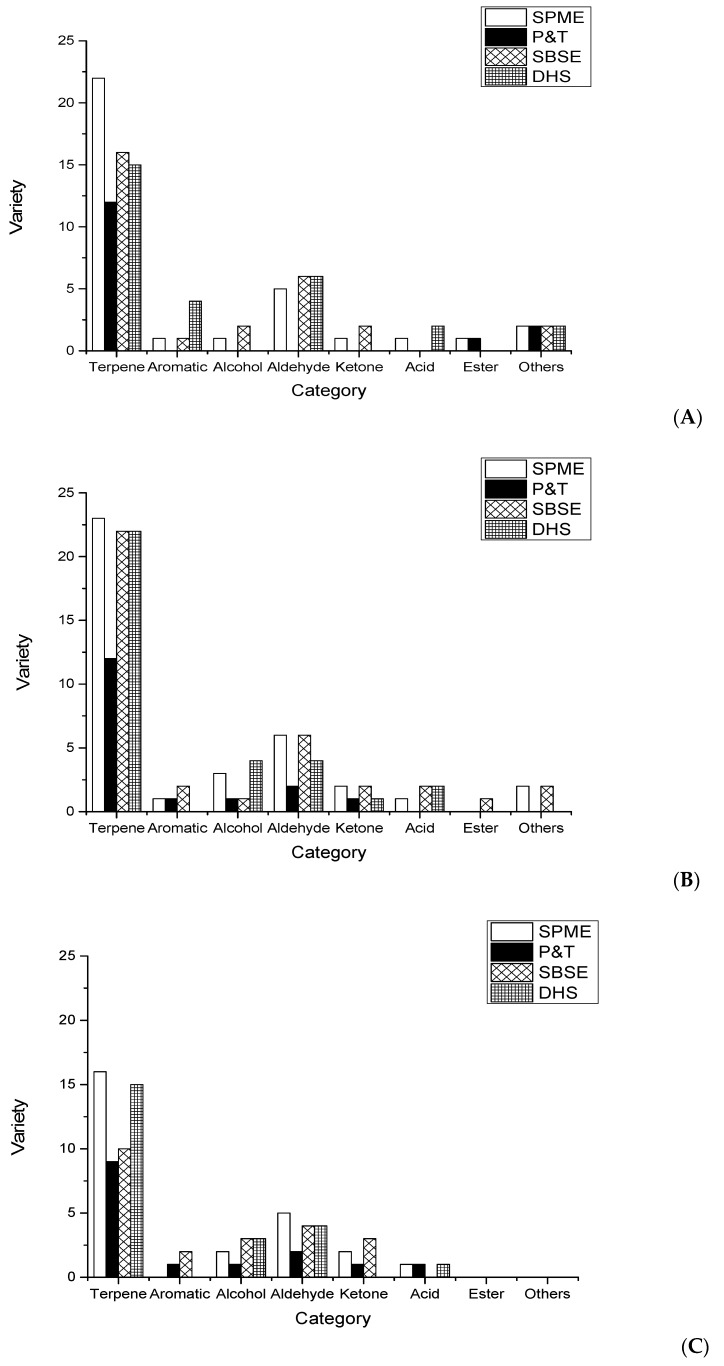
Quantities of the different kinds of flavor compounds extracted by SPME, P&T, SBSE, and DHS from the three kinds of *A. dahurica*: (**A**) Hangbaizhi, (**B**) Qibaizhi, and (**C**) Bobaizhi.

As shown in Figure 2, the volatile compounds extracted by the four different headspace extraction methods were different. P&T was unable to extract aromatics, alcohols, aldehydes, ketones, and acids from Hangbaizhi but extracted those compounds from Qibaizhi and Bobaizhi in low amounts. However, the amounts of these aroma compounds extracted from the three kinds of *A. dahurica* using SPME, SBSE, and DHS were all higher than those obtained using P&T. This result indicated that although these aroma compounds were present in Hangbaizhi, P&T may not be a suitable method for extracting the aroma-active compounds from *A. dahurica*. In addition, SPME, SBSE and DHS effectively extracted the terpenes and aldehydes. However, these three methods were not ideal for the extraction of aromatic species, alcohols, ketones, acids, and esters. Although there may have been low contents of these compounds in the samples, it is likely that the headspace extraction method may not effectively extract all types of aroma compounds; thus, incorporating a solvent extraction step into the method may be necessary. The SPME method is simple and consumes a small amount of material, while the SBSE and DHS methods require cold trapping technology, which not only consumes a larger amount of material but is also expensive. Thus, future research focusing on the extraction of aroma-active compounds from the roots of *A. dahurica* should be carried out using the SPME method.

### 2.3. Key Aroma-Active Compounds Identified by SPME-AEDA

To further explore the key aroma-active compounds in the root of *A. dahurica*, dilution analysis was performed using SPME in conjunction with AEDA. As shown in Table 2, there were 12, 16, and 15 compounds that contributed to odors of in Hangbaizhi, Qibaizhi, and Bobaizhi, respectively, as determined by the sniffing test. The FD factor reflects the magnitude of the contribution of each aroma compound: A high FD factor corresponds to a greater contribution to the flavor of *A. dahurica*.

As shown in Table 2, the FD value of prenol (herbal) was higher in Hangbaizhi (FD = 81) than in the other samples. Nonanal (citrusy) and α-copaene (woody) (FD = 27), as well as acetic acid (sour) and β-pinene (pine) (FD = 9), contributed substantially to the flavor of Hangbaizhi. Hence, these five compounds were key aroma-active compounds in Hangbaizhi. Sixteen aroma-active compounds were detected in Qibaizhi by the sniffing test. Prenol (herbal) (FD = 81), (−)-β-elemene (spicy) (FD = 81), β-pinene (pine) (FD = 27), γ-muurolene (herbal) (FD = 27), acetic acid (sour) (FD = 9), caryophyllene (woody) (FD = 9), and humulene (woody) (FD = 9) were key aroma-active compounds in Qibaizhi. Fifteen aroma compounds, including α-gurjunene (woody) (FD = 81), prenol (herbal) (FD = 81), α-copaene (woody) (FD = 27), nonanal (citrusy) (FD = 27), 2-methyl-2-butenal (fruity) (FD = 9), acetic acid (sour) (FD = 9), aromadendrene (woody) (FD = 9) and β-pinene (pine) (FD = 9), were identified in Bobaizhi, indicating that they were key aroma-active compounds in Bobaizhi. Compounds with FD ≤ 3 were considered to be minor contributors to the overall aroma, but these compounds did enhance the complexity of *A. dahurica* in some ways.

SPME-AEDA indicated that the key aroma-active compounds in the three types of *A. dahurica* were similar; however, some compounds had different FD factors. For example, the FD value of 2-methyl-2-butenal (fruity) (FD = 9) in Bobaizhi was higher than those in Hangbaizhi (FD = 1) and Qibaizhi (FD = 1). The FD values of β-pinene (pine) (FD = 27), (−)-β-elemene (spicy) (FD = 81), and caryophyllene (woody) (FD = 9) in Qibaizhi were higher than those in Hangbaizhi (FD = 9, 1, and 3 for β-pinene, (−)-β-elemene and caryophyllene, respectively) and Bobaizhi (FD = 9, 1, and 1, for β-pinene, (−)-β-elemene and caryophyllene, respectively). The FD value of nonanal (citrusy) (FD = 1) in Qibaizhi was lower than those in Hangbaizhi (FD = 27) and Bobaizhi (FD = 27). Among all, the intensity of the volatile compounds on the *A. dahurica* samples have not been studied yet.

As shown in Table 3, the external standard method was used to accurately quantify ten key aroma-active compounds shared by Hangbaizhi, Qibaizhi, and Bobaizhi as identified by SPME-AEDA. It can be seen from the table that the same aroma compounds had different FD values in the different *A. dahurica* cultivars due to the difference in their concentrations. These common aroma-active compounds provided the three kinds of *A. dahurica* with similar odor properties, but because of their different concentrations in each sample, they contributed to the overall odors to different extents. In addition to these ten common aroma compounds, the three kinds of *A. dahurica* each had several unique aroma-active compounds. For example, γ-muurolene (herbal) and humulene (woody) were key aroma-active compounds characteristic of Qibaizhi, and α-gurjunene (woody) was an aroma-active compound unique to Bobaizhi. These differences may have caused differences in the overall odors of these three kinds of *A. dahurica*.

### 2.4. Sensory Evaluation

**Figure 3:**
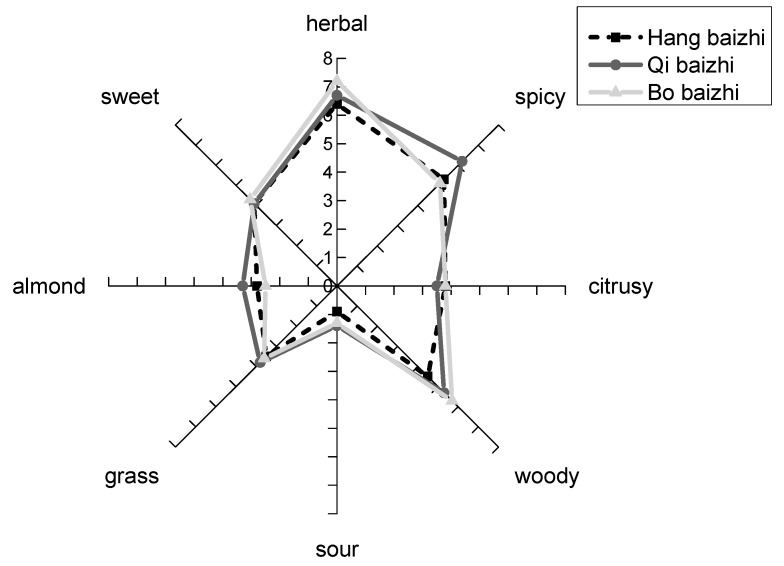
Odor sensory profiles of the three kinds of *A. dahurica*.

The sensory evaluation data shown in Figure 3 indicate that the main characteristic aromas of the three types of *A. dahurica* were herbal, spicy and woody. All three types of *A. dahurica* had high scores for herbal aroma. As indicated by GC-O, the herbal aroma was due to prenol, caryophyllene oxide, and γ-muurolene. The spicy aroma in Qibaizhi was more intense than that in the other samples due to the presence of (−)-β-elemene, as indicated by GC-O. The woody aroma of Bobaizhi had a higher score than that of the other samples; α-gurjunene, α-copaene and aromadendrene contributed to the woody odor of Bobaizhi. The contents of the woody flavor compounds in Hangbaizhi and Qibaizhi were lower. Other low-scoring aromas, such as citrus, were generated by other active-aroma compounds. This conclusion was based on the presence of these aroma-active compounds and their various aromas, which were detected in the sniffing tests, and thus these compounds contributed to the complex aroma of *A. dahurica*.

### 2.5. PCA and PLS-DA of the Aroma Compounds of A. dahurica

**Figure 4:**
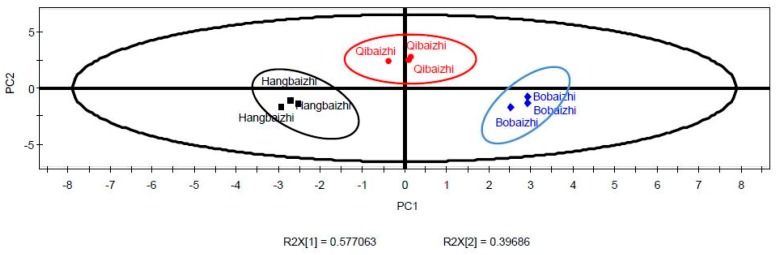
Scores plot of aroma-active compounds of *A. dahurica* by principal component analysis.

PCA and PLS-DA were performed to determine the differences in the key aroma compounds in the three kinds of *A. dahurica*. The concentrations of the ten shared key aroma compounds were used for PCA and PLS-DA. PCA score plots provide useful information about clustering samples into groups [16]. As shown in Table 4, three principal components were extracted, and these components represented 99.1% of the variation. The first, second and third main factors explained 57.7%, 39.7%, and 1.72% of the total variance, respectively; PC1 and PC2 were sufficient to explain the maximum variation in all the original samples of *A. dahurica* with a combined contribution of 97.4%. Figure 4 shows the PCA score plots of *A. dahurica*. The Hangbaizhi was located on the left while the Bobaizhi was located on the wright side, and the Qibaizhi was the upper middle portion of the correlation plot, indicating that they have the difference on aroma, three different kinds of *A. dahurica* can be clearly divided into three groups.

**Figure 5:**
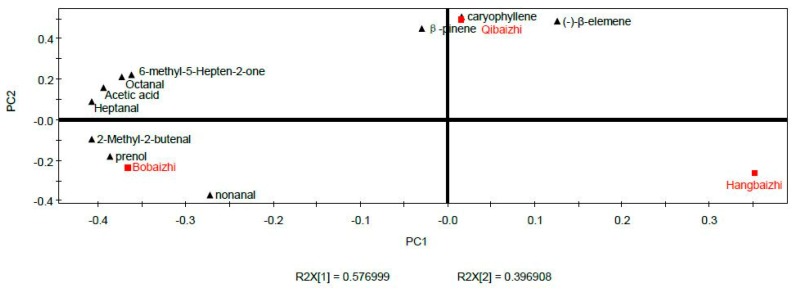
Loading plot of aroma-active compounds of *A. dahurica* by Partial least squares discriminant analysis.

PLS-DA is a chemometric technique used to optimize the separation between different groups of samples; its loading plots reflect the relationships among important variables specific to the group of interest, and variable importance on projection (VIP) can be used to identify the most important variables [17,18]. The resulting loading plot (PC1vs PC2) is shown in Figure 5, for the investugation of relationships of the aroma compounds and classification of Baizhi, the aroma compounds β-pinene (No.2), (−)-β-elemene (No.9) and caryophyllene (No.10) were clustered with Qibaizhi (class 2); which were located at the same area, and it was indicated that these compounds were positive correlation with Qibaizhi. The seven aroma compounds such as the 2-methyl-2-butenal (No.1), prenol (No.5), etc., related to Bobaizhi, were in the lower right portion of the diagram.

## 3. Materials and Methods

### 3.1. Preparation of A. dahurica

Three types of dry *A. dahurica* root, including Hangbaizhi, Qibaizhi, and Bobaizhi root, were purchased from Beijing Tongrentang and were identified as genuine products by Professor Meng Xiansheng of the Liaoning University of Traditional Chinese Medicine (Dalian, China). Prior to analysis, each sample was ground into a powder using an electric grinder and then passed through a 40-mesh sieve. The samples were kept at room temperature. The sieved powder was transferred to a sealed bag and then stored in a dark dry place.

### 3.2. Chemicals

*N*-Alkanes (C7–C30) used to calculate the retention indices (RI values) were obtained from Sigma-Aldrich (Milwaukee, WI, USA). All standards (β-pinene, (−)-β-elemene, prenol, 2-methyl-2-butenal, heptanal, octanal, nonanal, 6-methyl-5-hepten-2-one, caryophyllene, and acetic acid) used for qualitative and quantitative analysis of the aroma compounds were purchased from Sigma-Aldrich. Nitrogen (99.9992% purity) and helium (99.999% purity) were purchased from Beijing Haipubeifen Gas Industry Co. Ltd. (Beijing, China). Liquid nitrogen (99.99% purity) was purchased from Beijing Xianheyu Gas Industry Co. Ltd. (Beijing, China).

### 3.3. Extraction of the Volatile Compounds

Four different headspace extraction methods (SPME, P&T, SBSE, and DHS) were employed to isolate the volatile compounds from *A. dahurica*. The extracted volatile compounds were then subjected to GC-O-MS analysis, and the results were compared.

#### 3.3.1. Extraction of the Aroma Compounds from *A. dahurica* by SPME

SPME was carried out according to the method described by Lee, Cho, and Lee, K.G. [19] with minor modifications. Three grams of *A. dahurica* was weighed in a 40-mL odor-free headspace vial. The volatile compounds were extracted at 60 °C for 40 min with an equilibration time of 20 min by static SPME using 1-cm fibers coated with 50/30 μm divinylbenzene/carboxen on polydimethylsiloxane (DVB/CAR/PDMS) (57329-U; Supelco, Bellefonte, PA, USA). Then, the fibers were subjected to analysis by GC-O-MS at a temperature of 250 °C for 5 min. Each experiment was conducted in triplicate.

#### 3.3.2. Extraction of the Aroma Compounds from *A. dahurica* by P&T

The extraction by P&T was conducted following the method described in Cai and Huang [20] with minor modifications. Three grams of *A. dahurica* was weighed in a 40-mL odor-free headspace vial. The extraction conditions were as follows: purge temperature, 60 °C; equilibration time, 20 min; purge time, 40 min; flow rate of nitrogen, 50 mL/min; desorption temperature, 250 °C; and desorption time, 2 min. Then, the extracted volatile compounds were subjected to analysis by GC-O-MS. Each experiment was conducted in triplicate.

#### 3.3.3. Extraction of the Aroma Compounds from *A. dahurica* by SBSE

SBSE was carried out according to the method described by Hoz, Salinas, and Ferrandino [15] with minor modifications. Three grams of *A. dahurica*, weighed into a 40-mL odor-free headspace vial, was dissolved in 20 mL of ultrapure water. The extraction was conducted at 60 °C, and the sample was equilibrated for 20 min. HS-SBSE was carried out by exposing a commercial polydimethylsiloxane (PDMS)-coated stir bar to the sample in a hermetically sealed headspace vial, and the sample was stirred at 500 rpm and heated to 60 °C for 40 min. After that, the stir bar was rinsed with pure water and then dried with a lint-free tissue. The sample introduction system was composed of an automated thermal desorption unit (TDU) combined with a multipurpose sampler (MPS) and a cold injection system (CIS). The sample was analyzed using GC-O-MS. Each experiment was conducted in triplicate.

The heating program for the TDU was as follows: The initial temperature of 50 °C was held for 1 min, then it was increased to 200 °C at 60 °C/min and held for 10 min. Finally, the temperature was increased to 300 °C at 60 °C/min and held for 10 min.

The heating program for CIS was as follows: The temperature of the CIS was decreased to −100 °C using liquid nitrogen and then held until analysis using the TDU was completed. After that, the temperature was increased to 240 °C at 10 °C/min and held for 1 min.

#### 3.3.4. Extraction of the Aroma Compounds from *A. dahurica* by DHS

The DHS analysis was performed following the method described by Waehrens and Zhang [21] with minor modifications. Ten grams of *A. dahurica* was weighed in a 150-mL odor-free dynamic headspace bottle and placed in a circulating water bath at 60 °C for 20 min. After that, the sample was purged with high-purity nitrogen at a flow rate of 100 mL/min and then dynamically absorbed onto a Tenax column for 40 min. The volatile components adsorbed on the Tenax column were then injected through an MPS multisampler, where they were separated and analyzed by a thermal desorption system (TDU), a cold injection system (CIS), and GC-O-MS. Each experiment was conducted in triplicate.

The conditions for the TDU and CIS were consistent with those used in the SBSE method.

#### 3.3.5. GC-O-MS Analysis

In this work, GC-MS/MS (7890A-7000B; Agilent Technologies Inc., Santa Clara, CA, USA) was employed to analyze the aroma compounds, and a Sniffer 9000 Olfactometer (Brechühler, Schlieren, Switzerland) was used to detect their aromas. A DB-Wax (strong polarity, 30 m × 0.25 mm × 0.25 μm; J&W Scientific, Folsom, CA, USA) and DB-5 (weak polarity, 30 m × 0.25 mm × 0.25 μm; J&W Scientific, Folsom, CA, USA) column were used to separate the volatile compounds. The method was conducted according to Song and Liu (2018) with minor modifications. Ultrahigh-purity helium was used as the carrier gas at a flow rate of 1.2 mL/min. The heating program was as follows: The initial temperature was set at 40 °C and held for 3 min, then it was increased to 200 °C at a rate of 5 °C/min and finally raised to 230 °C at a rate of 10 °C/min and held for 3 min. The split ratio was 5:1, and the injector and GC-MS/MS interface temperatures were set at 250 and 280 °C, respectively. Electron impact mass spectra were generated at 70 eV, and the m/z scan was carried out from 50 to 350 m/z. The ion source temperature was set at 230 °C.

GC-O was carried out by five experienced assessors. The assessors smelled the aromas with sniffing masks and then recorded the aroma they perceived; the retention time, intensity value, and description of the aroma were all noted. Each assessor sniffed each sample twice. The compounds were considered to be aroma active when odors with the same retention time were detected by at least half of the assessors [22].

### 3.4. Qualitative Analysis of the Volatile Compounds in the Root of A. dahurica

The chemical identification was conducted by comparison to a database of mass spectra and the linear RI values reported in the literature, and the aromatic characteristics were established from references and sniffing tests. Some key aroma-active compounds were also revalidated by comparing their profiles to those of standard compounds. The RI values were calculated as follows based on a normal alkane series and comparison with references:

RI = 100 *n* + 100 *n* (t_a_ − t*_n_*)/(t*_n_*_+ 1_ − t*_n_*)

where ta represents the retention time of sample a, t*_n_* represents the retention time of the normal alkane series, and *n* is the number of carbon atoms [23].

### 3.5. SPME-AEDA

SPME-AEDA is similar to the method described by Kim et al. [24], in which dilution analysis can be achieved by changing the split ratio in the GC-O method. In this study, the split ratios of the GC-O method were 5:1, 15:1, 45:1, 135:1, and 405:1, which corresponded to FD factors of 1, 3, 9, 27, and 81, respectively. The assessors sniffed the effluents from the separations with different split ratios through sniffing masks, and the process was stopped when an aroma could not be detected; the results are expressed as the FD factor. The FD factor is related to the intensity of the odor and thus can indicate the contribution of a compound to the overall smell.

### 3.6. Quantitative Analysis of the Key Aroma Compounds in the Root of A. dahurica

In this study, the external standard method was used to accurately quantify the key aroma-active compounds shared by the three *A. dahurica* root samples analyzed by SPME-AEDA. The method used in this work was that described by Tominaga and Dubourdieu [25] with some modifications. The aroma compounds in the roots of *A. dahurica* were first extracted by SPME, and then, the key aroma compounds were analyzed by GC-O-MS in selective ion mode (SIM). The roots of *A. dahurica* were used as a matrix, and a mixed standard was added at different concentrations. Then, SPME was used for extraction, and GC-O-MS in SIM was used to analyze the key aroma compounds. The concentration of the compound to be quantified in the mixed standard was plotted along the *x*-axis, and the difference between the peak area of the matrix spiked with the mixed standard and that of the blank matrix was plotted along the *y*-axis. By adjusting the concentration of the external standards added to the sample, a standard curve was prepared. The compounds were accurately quantified using the standard curves. The standard curves of these compounds were calibrated by subtracting the blank ratios (the area of the peak of a selected ion of the compound found naturally in the root of *A. dahurica*).

### 3.7. Sensory Evaluation

Three grams of the *A. dahurica* sample was accurately weighed in a 40-mL headspace bottle. The sensory evaluation was carried out in a quiet, tasteless sensory evaluation room by ten trained assessors (6 women and 4 men with an average age of 25 years old from the Laboratory of Molecular Sensory Science, Beijing Technology and Business University). The evaluators did not consume food for at least one hour prior to evaluation, and during the evaluation, they were not allowed to communicate with each other. The interval time between each sample was 5 min. The intensity of each aroma was evaluated on a 10-point intensity scale: 1 indicated a weak intensity, 5 indicated a moderate intensity, and 10 indicated a strong intensity [26].

### 3.8. Statistical Analysis

PCA and PLS-DA were conducted using SIMCA-P+ 11.0 software to classify the three kinds of *A. dahurica* and identify the key aroma-active compounds that most influenced the classification.

Tables were constructed using Microsoft Word 2010. Figures were constructed using Origin software (version OriginPro 9.1, OriginLab Inc., USA). The analyses of the volatile compounds of each sample using SPME, P&T, SBSE, and DHS were carried out in triplicate.

## 4. Conclusions

It could be seen from this study that using SPME methods, the 34, 38, and 26 aroma compounds were extracted from Hangbaizhi, Qibaizhi, and Bobaizhi, indicating that they were more efficient methods than the other three extraction methods. Following this, the key aroma compounds were identified by SPME-AEDA, which were 20, 21, and 17 aroma-active compounds from these three samples, respectively. The Prenol, nonanal, α-copaene, acetic acid, and β-pinene were the key aroma compounds in Hangbaizhi; prenol, (−)-β-elemene, β-pinene, γ-muurolene, acetic acid, caryophyllene, and humulene were the key aroma compounds of Qibaizhi; and α-gurjunene, prenol, α-copaene, nonanal, 2-methyl-2-butenal, acetic acid, aromadendrene, and β-pinene were the key aroma compounds in Bobaizhi. Sensory evaluation combined with GC-O indicated that the aromas of Baizhi included herb, spice, and woody. PCA and PLS-DA also showed that the three different kinds of Baizhi can be divided into three groups on the basis of the differences in the concentrations of their key aroma-active compounds, and caryophyllene, (−)-β-elemene, nonanal, and β-pinene play important roles in the classification.

This study systematically explored the differences in the flavor compounds of *A. dahurica* root samples originating from three different locations, key aroma compounds of each *A. dahurica* root sample, and the flavor characteristics of the different *A. dahurica* samples, providing a theoretical basis for its use as a spice. In future, studies are needed to investigate the reason that leads to the different flavor characteristics of different samples and to analyze the pathways of the aroma compounds.

## Figures and Tables

**Table 1:** Qualitative evaluation of the aroma compounds collected from *A. dahurica* using solid-phase microextraction (SPME), purge and trap (P&T), stir bar sportive extraction (SBSE) and dynamic headspace sampling (DHS).

No	Compound	Odour ^1^	RI ^2^		Extract Method/Identification ^3^		
			DB-Wax	DB-5	Hangbaizhi	Qibaizhi	Bobaizhi
**Terpenes**							
1	α-Pinene	pine, turpentine	1032	939	SPME, SBSE, DHS/MS, RI	SPME, SBSE, DHS/MS, RI	SBSE, DHS/MS, RI
2	Camphene	camphor	1075		P&T, DHS/MS, RI, O	P&T, SBSE, DHS/MS, RI, O	P&T/MS, RI, O
3	β-Pinene	pine, resin	1116	981	SPME, P&T, SBSE, DHS/MS, RI, O, STD	SPME, P&T, SBSE, DHS/MS, RI, O, STD	SPME, P&T, SBSE, DHS/MS, RI, O, STD
4	Sabinene	turpentine, woody	1123	972	SBSE/MS, RI	SPME, P&T, SBSE, DHS/MS, RI	-
5	β-Myrcene	balsamic, spice	1145	992	SPME, SBSE, DHS/MS, RI	SPME, DHS/MS, RI	-
6	α-Phellandrene	turpentine, spice	1166		SPME, P&T/MS, RI	DHS/MS, RI	-
7	α-Terpinene	lemon	1178		-	SPME, SBSE/MS, RI	-
8	d-Limonene	citrus, mint	1201	1030	SPME, P&T, SBSE, DHS/MS, RI	SPME, P&T, SBSE, DHS/MS, RI	SPME, P&T, SBSE, DHS/MS, RI
9	β-Phellandrene	mint, turpentine	1209		SPME, P&T, SBSE/MS, RI	SPME, P&T, SBSE, DHS/MS, RI	P&T, DHS/MS, RI
10	Eucalyptol	mint, sweet	1213		SBSE/MS, RI	P&T, SBSE/MS, RI	SBSE/MS, RI
11	γ-Terpinene	gasoline, turpentine	1238		SBSE/MS, RI	SPME, SBSE, DHS/MS, RI	-
12	trans-β-Ocimene	sweet, herb	1242	1038	SPME, P&T, SBSE, DHS/MS, RI	SPME, P&T, SBSE/MS, RI, O	SPME, P&T/MS, RI, O
13	*p*-Cymene	solvent, citrus	1261		SPME, SBSE/MS, RI	SPME, SBSE, DHS/MS, RI	P&T, SBSE, DHS/MS, RI
14	α-Copaene	woody, spice	1488		SPME, P&T/MS, RI, O	SPME, P&T, SBSE, DHS/MS, RI, O	SPME, P&T, DHS/MS, RI, O
15	Linalool	flower, lavender	1537		-	-	SBSE/MS, RI
16	β-Cubebene	citrus, fruit	1546		-	SBSE/MS, RI	-
17	Selina-5,11-diene	woody	1553		SPME/MS, O	-	-
18	Longifolene-(V4)	woody	1573		SPME, DHS/MS, O	-	-
19	(−)-β-Elemene	herb	1583	1398	SPME, P&T, SBSE, DHS/MS, RI, O, STD	SPME, SBSE, DHS/MS, O, STD	SPME, SBSE, DHS/MS, RI, O, STD
20	Caryophyllene	woody, spice	1594	1467	SPME, P&T, DHS/MS, RI, O, STD	SPME, P&T, SBSE, DHS/MS, RI, O, STD	SPME, P&T, SBSE, DHS/MS, RI, O, STD
21	Aromandendrene	woody	1600	1475	SPME, P&T, DHS/MS, RI	SPME, P&T, DHS/MS, RI	SPME/MS, RI, O
22	γ-Elemene	green, woody, oil	1636	1425	SPME, SBSE, DHS/MS, RI	SPME, SBSE, DHS/MS, RI, O	SPME, P&T, SBSE, DHS/MS, RI
23	cis-β-Farnesene	citrus, green	1648		SPME, SBSE/MS, RI	SPME/MS, RI	-
24	Humulene	woody	1663	1467	SPME, P&T/MS, RI, O	SPME, P&T, SBSE, DHS/MS, RI, O	SPME, DHS/MS, RI
25	γ-Muurolene	herb, woody, spice	1681	1475	SPME/MS, RI	SPME, SBSE, DHS/MS, RI, O	-
26	δ-Elemene	woody	1688	1340	SPME, DHS/MS, RI	SPME, P&T, SBSE, DHS/MS, RI	SPME, DHS/MS, RI
27	β-Selinene	herb	1711	1436	SPME, P&T, DHS/MS, RI	SPME, SBSE, DHS/MS, RI	SPME, DHS/MS, RI
28	δ-Cadinene	thyme, woody	1749		-	DHS/MS, RI	DHS/MS, RI
29	α-Gurjunene	woody, balsamic	1760		-	DHS/MS, RI	SPME, DHS/MS, RI, O
30	α-Guaiene	woody, balsamic	1801		-	SPME/MS, RI	-
31	γ-Selinene	woody	1803	1455	SBSE, DHS/MS, RI, O	-	SPME/MS, RI
32	β-Guaiene	woody, spice	1831	1483	-	-	SPME, DHS/MS, RI
33	Germacrene B	woody, earth, spice	1864	1562	SPME/MS, RI	SPME, DHS/MS, RI	SPME/MS, RI
34	Caryophyllene oxide	herb, sweet, spice	1962		SPME, SBSE/MS, RI, O	SPME, SBSE/MS, RI, O	SPME/MS, RI, O
35	Spathulenol	herb, fruit	2129		SBSE, DHS/MS, RI	SBSE/MS, RI	SBSE/MS, RI
**Aromatics**							
36	Toluene	paint	1042	773	DHS/MS, RI	-	-
37	Benzene, 1,3-dimethyl-	plastic	1150	802	DHS/MS, RI	-	-
38	*o*-Xylene	geranium	1183	888	DHS/MS, RI	-	-
39	α-*p*-Dimethylstyrene	citrus, pine	1414		-	-	P&T/MS, RI
40	α,3-Dimethylstyrene	cocoa	1430		-	P&T/MS, RI	-
41	Estragole	licorice, anise	1655	1200	-	-	SBSE/MS, RI
42	Anethole	spice	1809		-	SBSE/MS, RI	-
43	Isoelemicin	spice, flower	1944	1596	SPME, SBSE, DHS/MS, RI	SPME, SBSE/MS, RI	SBSE/MS, RI
**Alcohols**							
44	3-Buten-2-ol, 2-methyl-	herb	968		-	DHS/MS, RI	DHS/MS, RI
45	Prenol	herb	1127	779	SPME/MS, RI, O, STD	SPME, DHS/MS, RI, O, STD	SPME, P&T, DHS/MS, RI, O, STD
46	1-Hexanol	resin, flower	1360		-	P&T, DHS/MS, RI, O	-
47	Terpinen-4-ol	turpentine, nutmeg	1591	1179	SBSE/MS, RI	-	SBSE/MS, RI
48	Benzyl alcohol	sweet, flower	1865		-	SPME/MS, RI	SPME, SBSE, DHS/MS, RI
49	1-Dodecanol	fat, wax	1972	1577	SBSE/MS, RI	SPME, SBSE, DHS/MS, RI	-
50	1-Hexadecanol	wax, flower	2378		-	-	SBSE/MS, RI
**Aldehydes**							
51	Butanal, 3-methyl-	malt	910		DHS/MS, RI	-	DHS/MS, RI
52	Hexanal	grass, tallow, fat	1084	801	SPME, SBSE, DHS/MS, RI	SPME, P&T, SBSE, DHS/MS, RI, O	SPME, SBSE, DHS/MS, RI
53	2-Methyl-2-butenal	green, fruit	1101	753	SPME, DHS/MS, RI, O, STD	SPME, DHS/MS, RI, O, STD	SPME, DHS/MS, RI, O, STD
54	Heptanal	fat, citrus, rancid	1174	903	SPME, SBSE, DHS/MS, RI, O, STD	SPME, P&T, SBSE/MS, RI, O, STD	SPME, SBSE/MS, RI, O, STD
55	Octanal	fat, soap, lemon	1280	1006	SPME, SBSE/MS, RI, O, STD	SPME, SBSE, DHS/MS, RI, O, STD	SPME, P&T, SBSE, DHS/MS, RI, O, STD
56	(*E*)-2-Octenal	green, nut, fat	1345	1060	-	SBSE/MS, RI	-
57	Nonanal	fat, citrus, green	1385	1104	SPME, SBSE, DHS/MS, RI, O, STD	SPME, SBSE, DHS/MS, RI, O, STD	SPME, P&T/MS, RI, O, STD
58	Decanal	soap, orange peel	1484	1209	-	SPME/MS, RI	-
59	Benzaldehyde	almond, sugar	1495	960	DHS/MS, RI	-	-
60	(*E*)-2-Nonenal	cucumber, fat	1527	1162	SBSE/MS, RI	SBSE/MS, RI	SBSE/MS, RI, O
61	2,6-Octadienal, 3,7-dimethyl-, (*Z*)-	lemon	1667		SBSE/MS, RI	-	-
**Ketones**							
62	6-Methyl-5-hepten-2-one	pepper, mushroom, rubber	1336	974	SPME, SBSE/MS, RI, O, STD	SPME, SBSE/MS, RI, O, STD	SPME, SBSE/MS, RI, O, STD
63	2-Nonanone	hot milk, green	1388	1093	SBSE/MS, RI	SPME, P&T, SBSE, DHS/MS, RI	SPME, SBSE/MS, RI
64	Camphor	camphor	1491		-	-	P&T, SBSE/MS, RI
**Acids**							
65	Acetic acid	sour	1450		SPME, DHS/MS, RI, O, STD	SPME, SBSE, DHS/MS, RI, O, STD	SPME, P&T, DHS/MS, RI, O, STD
66	Hexanoic acid	sweat	1829	1019	-	DHS/MS, RI	-
67	Oleic Acid	fat	2430	2082	-	SBSE/MS, RI	-
68	Dodecanoic acid	metal	2517		DHS/MS, RI	-	-
**Esters**							
69	Vinyl acetate	sour	990		P&T/MS, O	-	-
70	ethyl-(*E*)-cinnamate	flower, honey	2097		SPME/MS, RI	-	-
71	γ-Decalactone	peach, fat	2103		-	SBSE/MS, RI	-
**Other Compounds**							
72	UN1	amaretto	1286		SPME, P&T, SBSE, DHS/O	SPME, SBSE/O	-
73	UN2	grass	1478		SPME, P&T, SBSE, DHS/O	SPME, SBSE/O	-

^1^ Odor description was obtained by the combination of Flavornet website search and actual sniffing. ^2^ Retention index (RI) for the odorant on DB-wax and DB-5 columns. ^3^ Methods of odorant identification included RI, MS, O, and STD, which represent the linear retention index, mass spectrum, odor properties and comparison to authentic standards by GC-O-MS. UN1, UN2 represent the compounds that could be sniffed by olfactometry but could not detected by mass spectrometry.

**Table 2:** Dilution analysis of *A. dahurica* using SPME combined with AEDA.

No	Compound	CAS	RI		Odor		FD	
			DB-Wax	DB-5		Hangbaizhi	Qibaizhi	Bobaizhi
1	2-Methyl-2-butenal	1115-11-3	1101	753	green, fruity	1	1	9
2	β-Pinene	127-91-3	1116	981	pine, resin, turpentine	9	27	9
3	Prenol	556-82-1	1127	779	herb	81	81	81
4	Heptanal	111-71-7	1174	903	fatty, citrusy, rancid	1	1	1
5	trans-β-Ocimene	3779-61-1	1242	1038	sweetie, herb	-	1	1
6	Octanal	124-13-0	1280	1006	fatty, soap, lemon	1	1	1
7	6-Methyl-5-hepten-2-one	110-93-0	1336	974	mushroom	1	1	1
8	Nonanal	124-19-6	1385	1104	citrusy, green	27	1	27
9	Acetic acid	64-19-7	1450		sour	9	9	9
10	α-Copaene	3856-25-5	1488		woody, spice	27	3	27
11	Caryophyllene	87-44-5	1594	1467	woody, spice	3	9	1
12	(−)-β-Elemene	515-13-9	1595	1398	spice	1	81	1
13	Aromadendrene	489-39-4	1600	1475	woody	-	-	9
14	γ-Elemene	29873-99-2	1636	1425	green, woody, oil	-	1	-
15	Humulene	6753-98-6	1663	1467	woody	-	9	-
16	γ-Muurolene	30021-74-0	1681	1475	herb, woody, spice	-	27	-
17	α-Gurjunene	489-40-7	1760		woody	-	-	81
18	Caryophyllene oxide	1139-30-6	1962		herb, sweetie, spice	3	3	1

**Table 3:** Quantitative determination of the key aroma-active compounds collected from *A. dahurica*.

No	Compound	Y	R^2^	Hangbaizhi		Qibaizhi		Bobaizhi	
				Concentration (ng/g)	FD	Concentration (ng/g)	FD	Concentration (ng/g)	FD
1	2-Methyl-2-butenal	y = 11,238x + 468,104	0.9981	124.13 ± 9.19 ^a^	1	179.65 ± 10.26 ^b^	1	304.83 ± 15.53 ^c^	9
2	β-Pinene	y = 8207.2x + 15,568	0.9904	20.63 ± 1.29 ^a^	9	26.59 ± 0.48 ^b^	27	21.37 ± 2.60 ^a^	9
3	Heptanal	y = 2918.3x − 29,754	0.9969	45.99 ± 2.35 ^a^	1	105.64 ± 6.88 ^b^	1	140.09 ± 5.67 ^c^	1
4	Octanal	y = 5022x + 33,019	0.9989	50.62 ± 2.61 ^a^	1	141.46 ± 13.48 ^b^	1	155.08 ± 7.19 ^b^	1
5	Prenol	y = 1560.4x + 10,598	0.9917	109.15 ± 6.42 ^a^	81	142.78 ± 20.28 ^a^	81	361.94 ± 22.46 ^b^	81
6	6-Methyl-5-hepten-2-one	y = 32,472x + 5788.4	0.9983	3.24 ± 0.09 ^a^	1	4.36 ± 0.22 ^b^	1	4.48 ± 0.13 ^b^	1
7	Nonanal	y = 7734.4x + 41,903	0.9985	80.44 ± 4.61 ^b^	27	67.37 ± 5.06 ^a^	1	104.88 ± 3.26 ^c^	27
8	Acetic acid	y = 29,396x + 8 × 10^6^	0.9952	148.03 ± 1.32 ^a^	9	286.38 ± 11.27 ^b^	9	331.44 ± 14.04 ^c^	9
9	(−)-β-Elemene	y = 15,828x − 354,790	0.9954	310.28 ± 13.09 ^b^	1	789.51 ± 21.95 ^c^	81	126.29 ± 0.84 ^a^	1
10	Caryophyllene	y = 26,536x + 71,101	0.9951	21.6 ± 2.46 ^a^	3	73.04 ± 1.71 ^b^	9	21.47 ± 0.71 ^a^	1

Values marked by the lower-case superscript letters (from “a” to “c”) within a line denote statistically significant differences (*p* < 0.05).

**Table 4:** Eigenvalues of the four principal components, their contributions, and the cumulative contribution.

Principal Component	Eigenvalue	Contribution (%)	Cumulative Contribution (%)
1	5.19	57.7	57.7
2	3.57	39.7	97.4
3	0.155	1.72	99.1
